# 2210: Anatomical substrates of cognitive fatigue in aging and in Parkinson’s disease

**DOI:** 10.1017/cts.2017.210

**Published:** 2018-05-10

**Authors:** Sarah Elizabeth Burke, Immanuel B. H. Samuel, Qing Zhou, Benzi Kluger, Catherine Price, Mingzhou Ding

## Abstract

OBJECTIVES/SPECIFIC AIMS: Identify objective neurological substrates of cognitive fatigue in Parkinson’s disease and in aging. METHODS/STUDY POPULATION: Structural and diffusion MRI. Behavioral assessments for aged adults and Parkinson’s disease. RESULTS/ANTICIPATED RESULTS: Gray and white matter deficits that correlate with deficits in the basal ganglia for fatigued Parkinson’s disease patients Versus anterior cingulate cortex in healthy aged adults with fatigue. DISCUSSION/SIGNIFICANCE OF IMPACT: Over 50% of patients with Parkison’s disease and 38% of healthy older adults suffer from cognitive fatigue. However, diagnostics are limited to subjective surveys and there are no treatments for either population. Therefore, objective measures are greatly needed for better diagnosis and development of treatment targets.

